# Diabetes Mellitus in a Patient With Lafora Disease: Possible Links With Pancreatic β-Cell Dysfunction and Insulin Resistance

**DOI:** 10.3389/fped.2018.00424

**Published:** 2019-01-16

**Authors:** Ramona C. Nicolescu, Sara Al-Khawaga, Berge A. Minassian, Khalid Hussain

**Affiliations:** ^1^Division of Endocrinology and Diabetes, Department of Pediatrics, University of Liège, Centre Hospitalier Régional de la Citadelle, Liège, Belgium; ^2^Division of Endocrinology, Department of Pediatrics, Sidra Medicine Outpatient Clinic, Doha, Qatar; ^3^Division of Neurology, Department of Pediatrics, University of Texas Southwestern, Dallas, TX, United States

**Keywords:** Lafora disease, *EPM2A*, *EPM2B/NHLRC1*, insulin resistance, diabetes, glycogen metabolism

## Abstract

Lafora disease (LD) is a rare autosomal recessive disorder characterized by progressive myoclonic epilepsy followed by continuous neurological decline, culminating in death within 10 years. LD leads to accumulation of insoluble, abnormal, glycogen–like structures called Lafora bodies (LBs). It is caused by mutations in the gene encoding glycogen phosphatase (*EPM2A)* or the E3 ubiquitin ligase malin (*EPM2B/NHLRC1)*. These two proteins are involved in an intricate, however, incompletely elucidated pathway governing glycogen metabolism. The formation of EPM2A and malin signaling complex promotes the ubiquitination of proteins participating in glycogen metabolism, where dysfunctional mutations lead to the formation of LBs. Herein, we describe a 13-years-old child with LD due to a *NHLRC1* (c.386C > A, p.Pro129His) mutation, who has developed diabetes mellitus and was treated with metformin. We discuss how basic mechanisms of LD could be linked to β-cell dysfunction and insulin resistance.

## Background

Lafora disease (LD; OMIM 254780) is a fatal autosomal recessive inherited disease characterized by progressive myoclonic epilepsy followed by continued neurological decline due to polyglucosan inclusion bodies (insoluble glucans) accumulation in brain and other peripheral tissues culminating in death within 10 years (1). LD is particularly frequent in countries with high rates of consanguinity such as Mediterranean, Southern India, and the Middle East. The disease is extremely rare with an estimated overall frequency of ~4 cases per million individuals globally (2, 3). LD is caused by mutation of two genes, *EPM2A* encoding the glucan phosphatase laforin (a dual specificity phosphatase), and *EPM2B/NHLRC1* encoding E3 ubiquitin ligase malin (2). *PRDM8* mutation has also been reported in a single family as an additional gene involved in LD associated with an earlier onset disease (4).

Laforin-malin complex regulates diverse cellular pathways, including ubiquitin-proteasome system and glycogen metabolism, where their defects in these processes lead to LD. The clinical symptoms of EPM2A and NHLRC1 gene mutation are similar; however, NHLRC1 mutation patients tend to live longer than the EPM2A gene mutation (5, 6). Protein targeting to glycogen (PTG), one of the regulatory subunits of protein phosphatase 1 targeting glycogen, modulates the protein phosphatase 1 (PP1) affinity to its substrates such as the glycogen synthase (GS) and phosphorylase (PH) (7). The laforin-malin complex suppress PTG activity by promoting its proteasomal degradation (7). In *Epm2b* knockout mice, reduced expression of malin E3 ubiquitin ligase leads to the formation of LBs and the accumulation of insoluble laforin (8).

**Figure 1:**
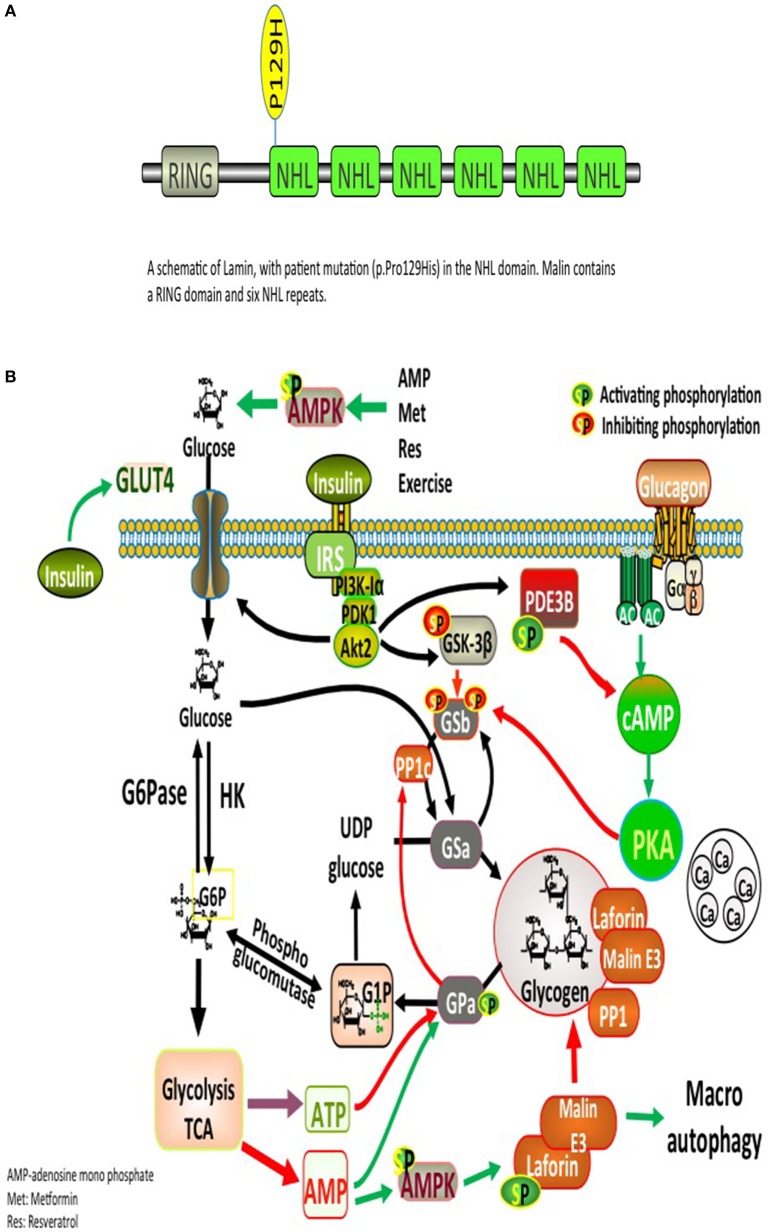
**(A)** Glycogen metabolism, degradation, and glycogen-metabolizing protein interactions. Both insulin and exercise increase glucose uptake via GLUT4. Increased Glucose-6-phosphate (G6P) levels provide feedforward activation of glycogen synthase (GS). HK, hexokinase; G6Pase, glucose-6-phosphatase; GNG, gluconeongenesis; PGM, phosphoglucomutase; GAA, lysosomal α-glucosidase; BE, branching enzyme; PH, glycogen phosphorylase; UP, UPD-glucose pyrophosphorylase; UGPPase, UDP-glucose pyrophosphatase; GN, glycogenin; GS, glycogen synthase; DBE, debranching enzyme; PKA, protein kinase A; LB, Lafora bodies (10). **(B)** A schematic illustration of Lamin structure with patient's mutation (p.Pro129His) in the NHL domain. Malin contains a RING domain and six NHL repeats.

Significant deposits of glucose in mammals occur in skeletal myocytes and hepatocytes, in addition to other tissues involved in glycogen synthesis, including the brain, cardiac myocytes, kidney, fat, and the pancreas (9). Glucose, lactate, alanine constitute the main precursors for glycogen synthesis. The direct pathway in glycogen synthesis requires transport of glucose into cells via the facilitative glucose transporters (GLUT) (10). Glycogenin self-glucosylates in a specialized initiation step, in which it can interact directly with GS to form α-1,4-glycosidic linkages of glycogen, utilizing UDP-glucose as the glucosyl donor. GS is allosterically activated by glucose 6-phosphate and negatively regulated by covalent phosphorylation (10). Glycogen-associated phosphatases (PP1Gs) composed of a catalytic subunit bound to a glycogen targeting subunit, dephosphorylates GS and other glycogen-metabolizing enzymes (11). PH and the debranching enzyme (DBE) AGL (amylo-α-1,6-glucosidase, 4-α-glucanotransferase) mediate glycogen degradation, to retrieve glucose in liver and muscle, in response to nutritional deprivation and exercise, respectively (10). A second pathway for glycogen degradation is through the lysosome and hydrolysis of glycogen to glucose by the lysosomal α-glucosidase (GAA) (10). A schematic illustration of glycogen metabolism and glycogen metabolizing protein interactions is displayed in Figure 1A.

Laforin and malin are involved in an intricate, however, incompletely elucidated pathway governing glycogen metabolism. Mutations in either protein result in the accumulation of glycogen into pathognomonic periodic acid-Schiff positive (PAS+) deposits named LBs that occur in many tissues, including retina, heart, liver, muscle, skin, and brain (2). LBs are abundant in all brain regions, specifically in the cell bodies of neurons and dendrites, but not abundant in axons, possibly elucidating on the cortical hyperexcitability seen in LD (2). The glycogen accumulated in LBs contains fewer α-1,6 branch points than normal glycogen, making it less branched and poorly soluble, similar to amylopectin found in plant starch, which explains its precipitation and time-dependent amassment into ever-enlarging LBs (2).

Laforin has several interacting partner proteins involved in glycogen metabolism, Among these interacting proteins are malin, PTG, GS, glycogen synthase kinase 3 (GSK3β), and EPM2A-interacting protein 1 (EPM2AIP1) (10). Other essential partners include a group of glycogen-associated PP1Gs constructed from PP1c bound to a glycogen-targeting subunits (1). Proper functioning of those interacting proteins plays a critical role in glycogen metabolism (Figure 1A). There is strong evidence suggesting that glycogen accumulation into LBs is a crucial process in the development of LD (12). Muscle glycogen synthase (MGS) is the dominant isoform expressed in the brain. Studies using malin and MGS knockout mouse model suggest that glycogen accumulation is the direct cause of the neurodegeneration and functional impairments seen in LD (12). Furthermore, in laforin and malin-deficient mice, knocking out protein phosphatase 1 regulatory subunit 3C (PPP1R3C) (also known as PTG; a protein involved in the activation of GS) prevented LBs formation, seizure susceptibility, and neurodegeneration (12). Herein, we shed light on possible mechanisms linking LD and diabetes.

## Case Presentation

A healthy 13-years-old boy presented with a tonic-clonic seizure, electroencephalography (EEG) demonstrated generalized spike-wave discharges, suggesting generalized epilepsy and sodium valproate was initiated as therapy. His anthropometric parameters at presentation were: weight 45 kg and height 160 cm (*Z*-score −0.6 and −0.4, respectively) with a body mass index (BMI) of 17.6 kg/m^2^ (Z-score 0.5). The clinical exam and the cognitive development were normal at time of presentation. By 6 months, the patient was on triple therapy (sodium valproate, perampanel, clonazepam) for increasing seizures and myoclonus. Six months following the diagnosis of epilepsy, he was found to have fasting (6.8 mmo/L) and varying postprandial (11.1–13.8 mmo/L) hyperglycemia, and glycosuria without ketonuria (Table 1). His past medical history was unremarkable, and he did not take other medications (apart from the antiepileptic medications) and had a negative family history for diabetes mellitus. His myoclonus worsened with progressive severe neurological sequelae (gait ataxia, loss of autonome ambulation, dysarthria, cognitive deterioration with extreme speech difficulties).

**Table 1:** Patient's clinical characteristics and treatment.

**Duration from diabetes diagnosis (months)**	**Clinical characteristics and biological profile**							**Treatment**	**Remarks**
	**Weight (kg) BMI (kg/m^**2**^)**	**Fasting blood glucose (mmol/L)**	**Insulin (mU/L)**	**C peptide (nmol/L)**	**HbA1c (%)**	**HOMA-IR**	**QUICKI**	
Diabetes diagnosis (6 months after 1st seizure episode)	45 BMI 17 (*Z* score 0.5)	6.8 13.8 (postprandial)	15.7	0.93	7.5	4.76	0.30	Insulin basal-prandial regimen 0.25 units/kg/d	Type of diabetes mellitus investigated
3 +	47 BMI 18 (*Z* score 0)				6			Long-acting insulin analog 0.2 units/kg/d	Continued insulin treatment (No diagnosis)
6 +^*^	–							Long-acting insulin analog 0.2 units/kg/d	Continued insulin treatment (No diagnosis)
12 +	48 BMI 17 (Z score −0.5)				6.5			Neither insulin, nor other diabetes treatment	No diagnosis
24 + (Lafora disease diagnosis)	50 BMI 17 (*Z* score −0.5)	12.21	29.4	1.62	8.2	15.97	0.26	Metformin 500 mg/d	Insulin resistance
36 +	52 BMI 17.3 (*Z* score −0.5)	11.1		1.8	8.3			Metformin 1,000 mg/d	Insulin resistance/T2DM
40 +	52 BMI 17.3 (*Z* score −0.5)	9.43	12	0.9	7.6	5.03	0.30	Metformin 1,500 mg/d	Insulin resistance/T2DM

+ *Autoimmune markers tested negative*.

* *No MODY mutations detected*.

*Reference range*.

*Insulin 2–17 mU/L*.

*C peptide 0.37–1.47 nmol/L*.

*HbA1c 4–6%*.

*T2DM, type 2 diabetes mellitus; HOMA-IR, homeostasis model assessment of insulin resistance 0.4–2.78; QUICKI, quantitative insulin sensitivity check index*.

Evaluation of glucose metabolism showed fasting hyperglycemia (6.8 mmol/L), glycosuria, negative ketonemia and ketonuria, and glycated hemoglobin (HbA1c) of 7.5%. The insulin secretion was preserved (15.7 mU/L, C peptide 0.93 nmol/L, fasting levels) with a HOMA-IR index (homeostasis model assessment insulin resistance) {calculated as [fasting glucose (mg/dl) x fasting insulin (lU/ml)/405]} at 4.76, suggestive of insulin resistance. The child did not display clinical features of insulin resistance (acanthosis nigricans, abnormal adipose tissue distribution, or lipodystrophy) and his lipid profile and hepatic function were normal at presentation and remained so during the follow-up.

The pancreatic autoimmune markers (ICA, GAD65, IAA, ZnT8) were negative. Testing for monogenic diabetes revealed no mutations in any of the known genes *(GCK, HNF1A, HNF4A, HNF1B, ABCC8, KCNJ11*, and *INS)*. The diagnosis of diabetes was made, a basal-prandial insulin regimen started, and a normal glycemic profile was quickly obtained with a very low total daily dose of insulin (0.25 μ/kg/d). The diagnosis of type 1 diabetes mellitus (T1DM) was not the right one, but at this point of diagnosis approach we were unable to define more precisely the association between the progressive myoclonic epilepsy and the hyperglycemia. Mitochondrial disease was also excluded.

Six months following the diagnosis of diabetes mellitus, the patient was under 0.2 μ/kg/d of long-acting insulin analog and showed an excellent glycemic profile (HbA1c 6%). Testing for autoimmune markers remained negative. A decision on continuing the same insulin regimen (only long-acting insulin analog) was encouraged.

Twenty four months following the first presentation and eighteen months after the diagnosis of diabetes, the patient's neurological status continued to worsen with a significant cognitive deterioration despite being under four antiepileptic drugs. His metabolic profile remained uncontrolled with persistent hyperglycemia (HbA1c 8.2%) and hyperinsulinemia (insulin 29.4 mUI/L, C peptide 1.62 nmol/L, fasting levels).

The complex nature of the metabolic and progressive neurological disease (uncontrolled seizures and unexplained insulin resistance) mandated high suspicion and testing for LD. PAS positive LBs are typically found in the eccrine duct and apocrine myoepithelial cells of sweat glands (13). An axillary skin biopsy was taken accordingly and revealing LBs within apocrine myoepithelium. Genetic testing displayed a homozygous mutation *NHLRC1* c.386C > A, p.Pro129His, confirming the diagnosis of LD (Figure 1B).

At the time of LD diagnosis, in the absence of any insulin regimen, a reevaluation of pancreatic insulin secretion demonstrated an increasing insulin resistance (insulin 29.4 mUI/L, C peptide 1.62–1.8 nmol/L, HOMA-IR 15.97). Based on the biochemistry results displaying high levels of endogenous insulin, metformin (starting a daily dose of 500 mg, increased to 1.5 g daily) was started with good tolerance and response (Table 1). As the progressive subsequent increase of HbA1c levels was noted, metformin was increased to 1,000 mg/day, which resulted in intestinal side-effects, and he was switched to long-acting insulin analog, without clear glycemic improvement (HbA1c at 8.3%). T1DM antibodies remained negative, with residual insulin secretion (C peptide at 1.8 nmol/l), confirming ongoing insulin resistance. The child did not develop clinical features of insulin resistance (acanthosis nigricans, abnormal adipose tissue distribution, or lipodystrophy). Currently, the patient remains under 1,500 mg metformin daily, with favorable glycemic control (Table 1). Due to the continuous neurological and clinical degradation, metformin administration was discontinued multiple times for several weeks resulting in fasting (13.8 mmol/L) and postprandial (16.6 mmol/l) hyperglycemia (Table 1).

## Discussion

We describe the clinical case of a 13-years-old child with genetically proven LD who has developed hyperglycemia, hyperinsulinemia, and insulin resistance (assessed by HOMA-IR and QUICKI indexes).

Novel autopsy findings have demonstrated pathologic findings of LBs diffusely deposited in several endocrine organs (14). Such results suggest that endocrine abnormalities should be considered in patients with LD. Herein, we discuss possible pathogenic links between LD and diabetes mellitus.

### How Does Laforin Act as a Regulator of Insulin Sensitivity?

Glycogen-targeting subunit (G_M)_ is the most commonly available glycogen-targeting subunit of PP-1 in rodent skeletal muscle. PP-1 plays an essential role in glucose metabolism through its regulatory effects on glycogen metabolizing enzymes, including GS, PH, and PH kinase. Homozygous G_M_^−/−^ mice demonstrated increased risk of obesity, glucose intolerance, and insulin resistance due to a significant decrease in the GS activity (15), suggesting that disruption of G_M_ may predispose to insulin resistance and diabetes. This observation is following the association of protein phosphatase 1 regulatory subunit 3 (PPP1R3/G_M_) and GS mutations with insulin resistance (16). Mice with heterozygous deletion of PTG, subunit R5 of PP1, or protein phosphatase 1 regulatory subunit 3C (PPP1R3C) develop glucose intolerance and insulin resistance (17). Although, the role of glycogen-targeting regulatory subunits (G subunits) in modulating the activities of the glycogen metabolizing enzymes through PP1-mediated dephosphorylation has been well-established, however, the role of G subunits in the regulation of postprandial glucose homeostasis remains largely unknown. Furthermore, the absence of the laforin interacting protein EPM2AIP1, which acts on GS, leads to hepatic insulin resistance through reduced allosteric activation of GS by glucose 6 phosphate (G6P) (18).

The *NHLRC1* mutation observed in our patient has been reported previously, but to date always in the compound heterozygous state (19). We speculate that the *NHLRC1* mutation in our patient might affect the laforin-malin-EPM2AIP1 complex formation in a mutation-specific manner and possibly lead to the insulin resistance phenotype. The insulin resistance manifested in our patient could be further attributed to disrupted malin function resulting in aberrant interaction with GS, PTG, and other glycogen-targeting regulatory subunits modulating glycogen metabolism. In conclusion, Epm2b–/– knockout mouse (20) was recently reported to display higher levels of glycogen in skeletal muscle, liver, and brain, which is also consistent with malin having a critical role in the regulation of glycogen metabolism (21). Furthermore, it was recently shown that disruption of the *Gys2* gene encoding the liver isoform of GS generates a mouse strain (LGSKO) characterized by an inability to synthesize liver glycogen with impaired hepatic insulin signaling and glucose disposal (22).

### Does β-Cell Dysfunction and Apoptosis Play a Role in LD?

The role of glycogen accumulation in β-cell dysfunction and apoptosis has been recently suggested (23). Chronic hyperglycemia leads to impaired oxidative metabolism and reduced ATP generation in response to glucose. Increased levels of G6P due to hyperglycemia stimulate GS and also lead to elevation of PPP1R3C (PTG), both of which promote glycogen accumulation. Hyperglycemia further participates in impaired autophagy; further increasing glycogen storage and enhancing β-cell death (23). Laforin-malin complex plays a vital role in regulating glycogen biosynthesis, a phenomenon consistent with the presence of the pathognomonic LBs inclusions. However, it is unclear whether the accumulation of LBs is the cause of the disease or whether they are secondary elements of a primarily established metabolic change (6). Increased glucose metabolism, rather than glucose *per se*, drives glycogen synthesis where impaired autophagy and increased cleaved caspase-3 suggest apoptotic pathways are also stimulated (23).

To our knowledge, this is the first report of an overt diabetes mellitus in a patient with LD. T1DM and monogenic diabetes were excluded (persistently negative pancreatic antibodies, preserved insulin and C peptide secretion, absence of MODY gene mutation). Mechanim(s) of the diabetes observed in our patient is (are) not completely clear yet. β-cell dysfunction and insulin resistance might contribute independently and intricately, as different mechanisms. This complex phenotype could be primarily attributed to the abnormal laforin-malin complexes and glycogen metabolism, ultimately leading to altered glucose homeostasis as seen in this pacient with LD.

### Study Limitations

We cannot precisely explain the triad of hyperglycemia, relative discrepancy between plasma insulin and glucose levels, and insulin resistance (confirmed by biochemistry data) observed in our patient. However, in the context of LD, this diabetes can be due to a combination of insulin resistance and β-cell dysfunction.

## Conclusion

Lafora disease is a rare autosomal recessive condition caused by mutations of either the *EPM2A* or the *EPM2B/NHLRC1* genes, encoding the laforin dual specificity phosphatase and the malin ubiquitin E3 ligase, respectively. Laforin-malin functional complex plays an essential role in glycogen metabolism. LD may be associated with hyperglycemia and overt diabetes. Our patient's metabolic evolution suggests that the pathophysiological mechanism of diabetes is a combination of insulin resistance and β-cell dysfunction. Our case suggests attention to metabolic profiles of current and future LD patients, and sheds light on possible links between insulin resistance/beta-cell dysfunction and Lafora disease due to dysfunctional lamin.

## Ethics Statement

Written informed consent for the presentation and publication of this case was obtained from the patient's parents.

## Author Contributions

RN and SA-K drafted the manuscript. RN, SA-K, KH, and BM have acquired, analyzed, and interpreted the data. All the authors have read the manuscript and have approved this article.

### Conflict of Interest Statement

The authors declare that the research was conducted in the absence of any commercial or financial relationships that could be construed as a potential conflict of interest.
